# Prospective, randomized, controlled trial assessing the effects of methylene blue for the prevention of hypotension during renal replacement therapy: protocol paper and statistical analysis plan for the BLUE study

**DOI:** 10.62675/2965-2774.20260300

**Published:** 2026-05-20

**Authors:** Carla Daniele Nascimento Pontes, Fernando Godinho Zampieri, Rodrigo Cruvinel Figueiredo, Rodolpho Augusto de Mouro Pedro, Luiz Marcelo Sá Malbouisson, Rodrigo Camillo da Cunha, Fernando Jose da Silva Ramos, Lucas Petri Damiani, Bruno Adler Maccagnan Pinheiro Besen, Flávio Geraldo Rezende de Freitas, Flávia Ribeiro Machado

**Affiliations:** 1 Universidade Federal de São Paulo Escola Paulista de Medicina Hospital São Paulo São Paulo SP Brazil Intensive Care Department, Hospital São Paulo, Escola Paulista de Medicina, Universidade Federal de São Paulo - São Paulo (SP), Brazil.; 2 Universidade Federal de São Paulo Escola Paulista de Medicina São Paulo SP Brazil Postgraduate Program in Translational Medicine, Escola Paulista de Medicina, Universidade Federal de São Paulo - São Paulo (SP), Brazil.; 3 Brazilian Research in Intensive Care Network São Paulo SP Brazil Brazilian Research in Intensive Care Network (BRICNet) - São Paulo (SP), Brazil.; 4 University of Alberta Department of Critical Care Medicine Alberta Canada Department of Critical Care Medicine, University of Alberta - Alberta, Canada.; 5 Centro Universitário do Espírito Santo Hospital São José Colatina ES Brazil Hospital São José, Centro Universitário do Espírito Santo - Colatina (ES), Brazil.; 6 Universidade de São Paulo Faculdade de Medicina Hospital das Clínicas São Paulo SP Brazil Intensive Care Unit, Department of Gastroenterology, Hospital das Clínicas, Faculdade de Medicina, Universidade de São Paulo - São Paulo (SP), Brazil.; 7 Universidade de São Paulo Faculdade de Medicina Department of Anesthesiology, Hospital das Clínicas São Paulo SP Brazil Intensive Care Unit, Department of Anesthesiology, Hospital das Clínicas, Faculdade de Medicina, Universidade de São Paulo - São Paulo (SP), Brazil.; 8 Hospital do Serviço Social da Indústria do Papel Papelão e Cortiça do Estado de São Paulo São Paulo SP Brazil Hospital do Serviço Social da Indústria do Papel, Papelão e Cortiça do Estado de São Paulo - São Paulo (SP), Brazil.

**Keywords:** Methylene blue, Hypotension, Hemodialysis, Acute kidney injury, Schock, Renal replacement therapy

## Abstract

**Objective:**

To describe the study protocol and statistical analysis plan that will be used to evaluate whether methylene blue reduces interventions aimed at controlling hypotension during renal replacement therapy as compared to usual care.

**Methods:**

BLUE is a randomized, multicenter, open-label trial. Patients with high risk of hypotension during renal replacement therapy will be randomized to receive either methylene blue infusion at a dose of 1mg/kg as a bolus, followed by continuous infusion of 0.1mg/kg of body weight in a total of 200mL of saline solution throughout the dialysis session, or to usual care. The usual care group will not receive any intervention. A total of 260 patients is expected to be randomized in a 1:1 ratio.

**Results:**

The primary outcome will be a composite of any of the following events: (1) initiation of vasopressor therapy or an increase of at least 20% from baseline dose; (2) interruption of the renal replacement therapy session; or (3) interruption of fluid removal at the request of the attending physician at any point during the session. Secondary outcomes include the occurrence of hypotension during the hemodialysis session, the maximum vasopressor dose within the first 24 hours, intensive care unit mortality, and in-hospital mortality.

**Conclusion:**

The BLUE study will provide evidence on the role of methylene blue in preventing hypotension during renal replacement therapy.

## INTRODUCTION

Renal replacement therapy (RRT) is widely employed in intensive care units (ICUs) for the management of acute or chronic kidney failure.^(1)^ Continuous RRT (CRRT) offers greater hemodynamic stability compared to conventional intermittent hemodialysis, particularly in hemodynamically unstable patients. However, its limited availability, high cost, and the absence of clear evidence of superiority over intermittent RRT regarding mortality or renal recovery hinder its widespread adoption – especially in resource-limited settings.^(2)^ As a result, intermittent RRT remains the most commonly used modality in these environments.

Intradialytic hypotension (IDH) is a frequent complication during intermittent RRT, with an incidence that may reach up to 70% in critically ill patients.^(3)^ Managing IDH poses a significant clinical challenge, as it is associated with increased morbidity and mortality, higher rates of catheter-related thrombosis, reduced likelihood of renal recovery, and impaired fluid and electrolyte control.^(4)^ Additional consequences include premature interruption of fluid removal, volume overload, and diminished dialysis efficiency.^(5)^

During hemodialysis, endothelial nitric oxide synthase activators may be upregulated through mechanisms not yet fully understood. Contact with dialysis membranes promotes cytokine release by monocytes, inducing tissue injury, increasing endothelial nitric oxide synthase activity, and, consequently, greater nitric oxide production.^(6)^ Endothelial nitric oxide plays a key role in cardiovascular regulation, particularly in blood pressure control. Excessive nitric oxide secretion can result in unwarranted vasodilation, leading to hemodynamic instability. The hypotensive effect and direct cytotoxicity of nitric oxide can lead to tissue hypoxia and multi-organ dysfunction.^(7)^

Methylene blue is a synthetic dye that acts as an inhibitor of the nitric oxide production pathway. Unlike conventional vasopressors, its effect occurs by inhibiting the nitric oxide-cGMP cascade, a central mechanism in RRT-related vasoplegia. This unique property supports its investigation as a preventive strategy against intradialytic hypotension or hemodynamic deterioration during hemodialysis sessions. Its main potential application would be as an adjunctive agent, reducing catecholamine requirements in the management of intradialytic hypotension.^(8)^

We hypothesize that methylene blue infusion can reduce the frequency of hypotension during RRT and, consequently, decrease unwarranted interruptions to this therapy. To our knowledge, no randomized clinical studies have used methylene blue during RRT in critically ill patients. To test this hypothesis, we designed this multicenter, randomized clinical trial to evaluate whether methylene blue infusion during RRT sessions, compared with usual care, reduces interventions aimed at controlling IDH.

The primary objective is to describe the study protocol and statistical analysis plan that will be used to evaluate whether methylene blue reduces interventions aimed at controlling hypotension during RRT as compared to usual care. The secondary objectives are to evaluate whether methylene blue reduces the incidence of hypotension, vasopressor dose requirements within the first 24 hours, ICU and in-hospital mortality, and adverse events.

## METHODS

### Study design and participating sites

This is a pragmatic, randomized, multicenter, open-label, controlled clinical trial. Critically ill patients admitted to the ICU who require RRT and who have a high risk of hypotension will be randomly assigned to receive methylene blue or usual care during the RRT session. The study protocol adheres to the SPIRIT 2013^(9)^ reporting guidelines.

The study will be conducted in five ICUs. The coordinating center is the *Hospital São Paulo* ICU at the *Universidade Federal de São Paulo* (UNIFESP). The other centers are general ICUs with a mixed population of clinical and surgical patients. All institutions have RRT management protocols implemented.

### Trial organization and oversight

An independent Data and Safety Monitoring Board (DSMB) will oversee the trial, ensuring safety, conducting an interim analysis, and determining whether to continue the study. The Research and Ethics Committee of the UNIFESP approved this study under the number 4.952.266, followed by approval from the *Hospital do Serviço Social da Indústria do Papel, Papelão e Cortiça do Estado de São Paulo* (SEPACO) Ethical Committee, the Ethics Committees of *Hospital São José*, and the *Hospital das Clínicas* of the *Faculdade de Medicina* of the *Universidade de São Paulo* (USP).

Written informed consent will be obtained from all participants or their legally authorized representatives before inclusion in the trial. The study is registered on the clinicaltrials.gov platform (identifier: NCT05092165) and is funded by a research grant from the *Conselho Nacional de Desenvolvimento Científico e Tecnológico* (CNPq).

### Eligibility criteria

Participants with a clinical indication for intermittent RRT - whether for acute or chronic kidney dysfunction and regardless of whether it is their first hemodialysis session - who are at high risk of developing hypotension will be eligible for inclusion. High risk of intradialytic hypotension is defined as the need for vasopressor support, adjusted at the discretion of the attending physician, or a systolic blood pressure below 100mmHg.^(10)^

Participants will be enrolled after meeting all inclusion criteria and none of the exclusion criteria, as outlined in table 1. Study sites will maintain continuous records of all patients who meet the inclusion criteria but are not enrolled, along with the reasons for non-inclusion, in accordance with the Consolidated Standards of Reporting Trials (CONSORT) guidelines.^(11)^

**Table 1:** Eligibility criteria

Inclusion criteria	Exclusion criteria
Patients over 18 years old Acute kidney injury or acute-on-chronic kidney disease requiring intermittent renal replacement therapy, hemodialysis Systolic blood pressure lower than 100mmHg or use of a vasopressor Signed informed consent	Pregnancy Life expectancy is less than 24 hours Exclusive palliative or end-of-life care Renal replacement therapy due to hypertensive crisis Known allergy to methylene blue Known glucose-6-phosphate dehydrogenase deficiency Prior participation in the study cute coronary syndrome Chronic nitrate oral use

RRT - renal replacement therapy.

### Randomization and allocation concealment

**Figure 1:**
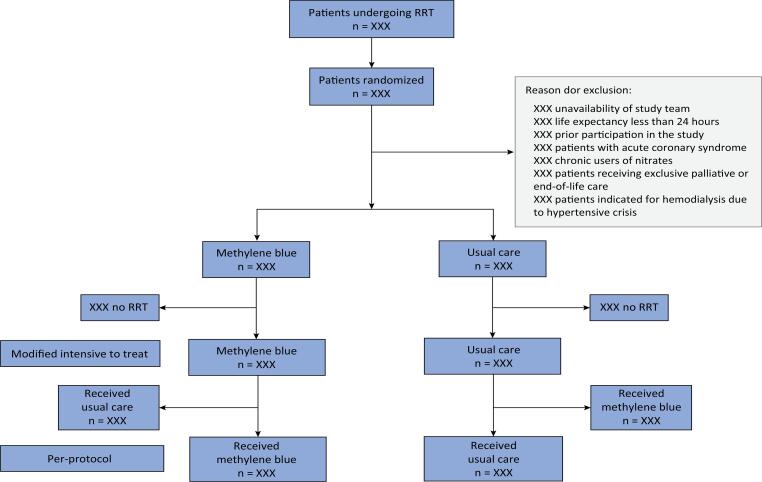
Participants’ allocation.

Participants will be randomized to receive either methylene blue or usual care through a centralized, web-based automated randomization system (RedCap® UNIFESP). The randomization list will be generated electronically on random.org by an external individual not involved in the study, using variable block sizes of 4 - 6, stratified by center and prior vasopressor use. Randomization access will be available 24 hours a day, 7 days a week. Each registration number will be randomized only once to prevent the same participant from being randomized more than once in the study. Randomization will be carried out automatically without the investigators’ knowledge. The participating centers were unaware of the block sizes to ensure allocation concealment. The flowchart is available in figure 1.

RRT - renal replacement therapy.

### Blinding

Blinding of healthcare professionals is not feasible due to methylene blue's distinct visual appearance. However, outcome assessors and statisticians will remain blinded to group allocation to minimize bias.

### Interventions

After meeting all study criteria, participants will be randomized to either the methylene blue group or the usual care group. Immediately before the RRT session begins, the BLUE group will receive an intravenous infusion of methylene blue. Following a previous study,^(12)^ 1mg/kg of methylene blue 2% diluted in 50mL of saline solution will be administered as a bolus over 5 minutes, followed by a continuous infusion of 0.1mg/kg body weight in a total of 250mL of saline solution throughout the RRT session.

Methylene blue will be produced and labeled by Magister Medicamentos Ltda ME (São Paulo, SP, Brazil). Once diluted in saline solution, it must be administered immediately.^(13,14)^ During the hemodialysis session in the methylene blue group, patients receive both a bolus and a maintenance infusion, with a total volume not exceeding 180 mL, to a maximum expected RRT duration limited to 6 hours. The standard care group will undergo RRT without receiving methylene blue. RRT sessions will follow institutional standards in both groups, including sodium profiling and dialysate temperature adjustments. Session duration may vary between 2 and 6 hours according to each participant's clinical needs. In both groups, participants will be continuously monitored using electrocardiography, pulse oximetry, and invasive or non-invasive blood pressure. Vital signs will be recorded every 15 minutes. Capillary blood glucose will be measured hourly.

Usual care will consist of standard hemodynamic management with fluids and vasopressors (norepinephrine as the first-line agent and vasopressin as the second-line agent), according to routine ICU practice. No additional intervention will be performed; patients in the control group will not receive methylene blue. Renal replacement therapy interruption and adjustments in ultrafiltration rate will be individualized and determined at the attending physician's discretion in both groups.

### Outcomes

The primary outcome will be a composite consisting of any of the following events: (1) initiation of vasopressor therapy or an increase in vasopressor dose by at least 20% from baseline; (2) interruption of the RRT session; (3) interruption of fluid removal at the request of the attending physician at any point during the session. No predefined interruption criteria were established due to the wide range of clinical scenarios encountered in patients undergoing RRT; thus, decisions regarding treatment interruption were left entirely to the attending physician's discretion and will not be adjudicated.

**Figure 2:**
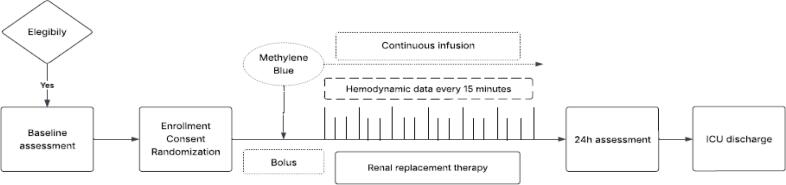
Study timeline.

Secondary outcomes include the occurrence of hypotension during the RRT session, the maximum vasopressor dose within the first 24 hours after the hemodialysis session, the cumulative norepinephrine equivalent (NEE) dose,^(15)^ the RRT session fluid balance, the 24-hour fluid balance, ICU mortality, and in-hospital mortality. Hypotension will be defined as a sustained drop in mean arterial pressure (MAP) below 65mmHg. We will assess potential adverse events related to methylene blue use, specifically: acute myocardial infarction, hemolytic anemia, cardiopulmonary arrest during the session, and worsening of gas exchange. We will present all outcome data as specified in table 1S (Supplementary Material). The trial sequence is available in figure 2.

ICU - intensive care unit.

### Data collection

Demographic characteristics, comorbidities, and severity of illness will be collected at baseline for all participants (Table 2S - Supplementary Material). Renal replacement therapy main characteristics and baseline hemodynamic data will be collected (Table 3S - Supplementary Material), and vital signs will be recorded every 15 minutes by the nursing team via invasive blood pressure or automated non-invasive blood pressure for the duration of the session, along with any changes in vasoactive drug use (Table 4S - Supplementary Material). We will collect variables assessed up to 24 hours, then participants will be followed until hospital discharge to determine survival status or death.

Participants who are withdrawn from the study for safety reasons (excluding those who withdraw consent) will be included in the analysis on an intention-to-treat basis. Any adverse events occurring within the first 24 hours after the hemodialysis session will be documented (Table 5S - Supplementary Material). Adherence to the intervention will also be recorded (Table 6S - Supplementary Material). The data collection schedule is presented in table 7S (Supplementary Material).

Data will be collected via electronic forms using the RedCap platform and securely stored. Forms will be completed by the investigators, who will use specific passwords to access them. Training will be provided for all personnel involved in the study to ensure high-quality data collection. The principal investigator will be available for any questions regarding form completion. Data consistency will be periodically reviewed, and any discrepancies will be addressed by contacting the sites for clarification and correction.

### Ethical considerations

This study was approved by the Research Ethics Committee of the UNIFESP under approval number 48165521.3.0000.5505, by the Research Ethics Committee of the *Hospital e Maternidade* SEPACO, by the Research Ethics Committee of the *Hospital São José*, and by the Research Ethics Committee of the *Hospital das Clínicas* of the *Faculdade de Medicina* of the USP. All participants or their legally authorized representatives will provide signed informed consent.

Participant inclusion will begin only after formal ethical approval. Investigators are committed to following international Good Clinical Practice requirements and the Declaration of Helsinki, and to adhering to national ethical standards set forth in the National Health Council Resolutions 466/2012 and 510/2016, ensuring the ethical conduct of the study and the safety of participants.

Only initials and hospital registration numbers will be recorded. All clinical and study-related information will be treated confidentially. Clinical records will be stored in areas with restricted access, limited to investigators and delegated data collection staff.

### Statistics

#### Sample size calculation and interim analysis

Based on a pilot study (data not shown), we expected the primary outcome to occur in 90% of the participants in the usual care group. Based on a previously published outpatient's clinic study, we expected an absolute reduction of 15% among those participants receiving methylene blue. Considering a 90% power and assuming a two-tailed alpha of 0.05, 130 participants would be required in each group.

An interim analysis will be performed after the inclusion of 100 participants to assess potential safety issues with the intervention regarding the primary outcome. The DSMB will review adverse event, compliance, and safety data. We do not plan to stop the study for futility or superiority. We plan to stop the study for safety using a stopping rule with a p-value < 0.01 for harm at the interim analysis. The DSMB will provide a recommendation based on the data, external evidence, and safety profile.

#### Statistical analysis

Our primary analysis will follow a modified intention-to-treat (mITT) approach, including all randomized participants who consent to participate. As clinical management might change after randomization, we expect that some participants randomized will not undergo RRT, either because of clinical improvement or deterioration. Thus, mITT analysis will include only those participants who received RRT. The data analysis will be performed after the submission of the protocol paper and analysis plan for publication.

Categorical variables will be expressed as counts and percentages. Continuous variables will be presented as means and standard deviations (SD) or medians with interquartile ranges (IQR, 25^th^ - 75th percentile), as appropriate. For all outcomes, including the primary outcome, we will not impute missing data, and the number of missing values will be clearly presented in tables. Treatment adherence will be reported as receiving the medication as a bolus, as a continuous infusion, or both.

The composite primary outcome will be measured using a mixed logistic model adjusted for non-linear terms of age, baseline MAP, vasopressor dose, and study site. Vasopressor dose will be defined as noradrenaline dose equivalents.^(15)^ A per-protocol analysis will be performed, including only participants who received the assigned medication. Additionally, we will conduct a sensitivity analysis that considers only participants who received the medication as a bolus plus continuous infusion. In all primary outcome analyses, the estimand will be the marginal risk difference between the intervention and control groups, along with their 95% confidence intervals, using the R package. Odds ratios will also be reported. Age and MAP will be included as restricted cubic splines in the model. In case any model does not converge, a stepwise approach will be used: (1) remove cubic splines; (2) remove the random effect for the site; (3) use Poisson or binomial regression.

Secondary outcomes will be assessed using similar models for categorical outcomes and using linear models for continuous variables, with adjustment for age, baseline MAP, vasopressor dose. For secondary binary and exploratory outcomes, logistic regression will be used, with the effect estimate expressed as marginal differences. The frequency of adverse events will be expressed as counts and percentages, and comparisons.

To demonstrate changes in physiological variables over time, we will present box plots. We will compare differences between groups in a mixed model, adjusting for age and baseline NEE, including participants as random effects, time and treatment as fixed effects, and treatment-by-time interactions.

For the primary outcome, we will perform a sensitivity analysis on the use or non-use of vasopressors at the initiation of dialysis therapy and at the first dialysis session *versus* non-use. We hypothesize that methylene blue will have more pronounced effects in patients without vasopressors and patients on their first dialysis session.

We will consider a significant two-tailed p level ≤ 0.05. Analysis will be performed using the current version of R (R Foundation for Statistical Computing, Vienna, Austria).

We do not expect participants to have missing outcome data for the primary composite outcome and the secondary outcome. If, for some reason, missing data occur because the researchers have not entered the data, we plan to perform multiple imputation. If a patient dies within the 24 hours time frame of the secondary outcomes, we will not make data imputations. For the physiological variables that will be compared over time, we will not make imputations, as the mixed model handles missing data as missing at random. If a patient dies within the 24 hours time frame of this data collection, we will use the last observation carried forward (as the most likely worst value of the physiological variable) to present these physiological analyses.

### Safety and adverse events

Given the severely ill ICU population who regularly undergo hemodialysis, we anticipate potential complications. These events will only be considered adverse events if they are potentially related to the study treatment, are unexpected to the attending physician, and are unrelated to the underlying critical illness that led to RRT.

Serious adverse events will be those that are life-threatening, result in death, lead to prolonged hospitalization, or cause severe and permanent disability. Serious adverse events will be adjudicated by independent physicians and reported to the DSMB and ethics committees. Details on adverse events are available in the notes.

Information on adverse events will be collected. As this involves a critically ill ICU population, various physiological alterations are expected, including laboratory abnormalities and vital sign changes, as well as those resulting from ongoing treatments. Therefore, expected abnormalities will not be considered adverse events unless there is suspicion or confirmation that the event is related to the study intervention.

Adverse events of special interest - those already known to be associated with methylene blue, regardless of severity - will also be recorded. These include hypertension (defined as systolic blood pressure above 180mmHg), chest pain, nausea, vomiting, gastric reflux (defined as reflux greater than 500mL within 6 hours after the start of dialysis), malignant hyperthermia (defined as the occurrence of hyperthermia associated with muscle rigidity, metabolic acidosis, hypercapnia, and/or rhabdomyolysis), hemolytic anemia (defined by the presence of anemia accompanied by clinical signs such as jaundice, together with laboratory evidence of red blood cell destruction such as elevated lactate dehydrogenase - LDH, increased indirect bilirubin, low haptoglobin, and/or reticulocytosis) and worsening of gas exchange, defined as a ≥ 20% decrease in the in partial pressure of oxygen in arterial blood/fraction of inspired oxygen (PaO_2_/FiO_2_) ratio as an absolute increase of 20% in FiO_2_ requirement, if blood gases were not available. In addition, any adverse events deemed by investigators to be possibly related to the study drug must also be documented. These data will be collected only within the first 24 hours following the drug infusion.

Serious adverse events will be defined as events resulting in one of the following outcomes: a) death; b) life-threatening situation; c) persistent or significant disability/incapacity; d) prolonged hospitalization; e) any suspected transmission of an infectious agent via a medicinal product; f) any clinically significant event.

Serious adverse events will be reported to the Research Ethics Committee (REC), in accordance with the guidelines of *Comissão Nacional de Ética em Pesquisa/Secretaria-Executiva do Conselho Nacional de Saúde/Ministério da Saúde* (CONEP/SECNS/MS) Circular Letter No. 13/2020. In the event of a reaction to the drug or an adverse event that the site investigator considers related to methylene blue, administration of the drug must be discontinued.

## DISCUSSION

This study protocol describes a randomized, open-label, pragmatic, multicenter study designed to evaluate the effects of methylene blue as an inhibitor of hemodialysis-related vasoplegia in critically ill patients. The primary outcome will be a composite of vasopressor use and modifications in the RRT.

Nitric oxide is the most potent endogenous vasodilator in humans, and its release during hemodialysis is triggered by metabolic stress and blood-membrane interaction. Its release during hemodialysis occurs in response to metabolic stress and inflammation caused by the contact between blood and the dialyzer membrane.^(16)^ In this context, the use of methylene blue is justified due to its inhibitory effect on the nitric oxide pathway, acting as an inhibitor of soluble guanylate cyclase, thereby reducing the effects mediated cGMP, which promotes vascular smooth muscle relaxation.^(17)^

It is believed that methylene blue may attenuate excessive vasodilation during hemodialysis and, consequently, reduce the incidence of intradialytic hypotension.^(16)^ If these beneficial effects are confirmed, the use of this agent could contribute to fewer interruptions of dialysis sessions, improved fluid removal, and better adherence to the treatment plan.^(18)^ Being a low-cost drug with few side effects, if we demonstrate its efficacy, it could enhance the safety of hemodialysis sessions in critically ill patients. This could have a significant impact on clinical practice, as intermittent techniques are the most common RRT available in limited-resource settings. It is of utmost importance to provide an appropriate answer to questions relevant to these settings.^(19)^

Our study design has several strengths. It will be conducted in ICUs that serve a diverse population of high-complexity medical and surgical patients. Additionally, randomization with allocation concealment through a centralized web-based system will help minimize selection bias. Our predefined primary and secondary outcomes are clinically relevant and directly related to patient safety and treatment efficacy. The protocol also includes a robust safety monitoring framework, with a DSMB overseeing study progress and ensuring participant safety.

We also recognize certain limitations. The main limitations of this study are the absence of long-term follow-up and the lack of patient-centered outcomes. Furthermore, tissue nitric oxide levels will not be measured, limiting our ability to determine whether the intervention effectively reduced systemic nitric oxide concentrations.^(20)^ The open-label design may introduce performance bias. Although blinding is not feasible for healthcare professionals administering the medication, efforts will be made to blind outcome assessors, data collectors, and statisticians, thereby minimizing the risk of bias in outcome evaluation.

### Role of sponsor source and conflicts of interest

This trial is investigator-led, and institutional funds provided the drug. Flávia Ribeiro Machado has a research grant from the CNPq. CNPq is not involved in the study design, data analysis, manuscript preparation, or decision to submit results for publication. The authors declare no conflicts of interest.

## CONCLUSION

The BLUE study is expected to offer valuable insights into the therapeutic potential of methylene blue in managing intradialytic hypotension, positioning it as a novel preventive strategy for this condition and its associated complications, with the potential to significantly improve patient outcomes.

## Data Availability

The study results will be disseminated through scientific communications, including presentations at medical congresses and publication in peer-reviewed journals. All principal investigators involved at the centers will be listed as authors, provided they meet international authorship criteria. Study data will be available from the authors upon reasonable request.
