# Supplementary material for: Prospective, randomized, controlled trial assessing the effects of methylene blue for the prevention of hypotension during renal replacement therapy: protocol paper and statistical analysis plan for the BLUE study
Source: Crit Care Sci. 2026 May 20;38:e20260300. doi: 10.62675/2965-2774.20260300 (PMC13399247; doi:10.62675/2965-2774.20260300)
Supplement: Supplementary Material [file 2965-2774-ccsci-38-e20260300-suppl01.pdf]

Critical Care Science
https://doi.org/10.62675/2965-2774.20260300

SUPPLEMENTARY MATERIAL

Prospective, randomized, controlled trial assessing the effects of methylene blue for the prevention of hypotension during renal replacement therapy: protocol paper and statistical analysis plan for the BLUE study

Carla Daniele Nascimento Pontes[1,2], Fernando Godinho Zampieri[1,3,4], Rodrigo Cruvinel Figueiredo[5], Rodolpho Augusto de Mouro Pedro[6], Luiz Marcelo Sá Malbouisson[3,6,7], Rodrigo Camillo da Cunha[8], Fernando Jose da Silva Ramos[1,2,3], Lucas Petri Damiani[1], Bruno Adler Maccagnan Pinheiro Besen[1,3], Flávio Geraldo Rezende de Freitas[1,2,3,8], Flávia Ribeiro Machado[1,2,3], and BRICNet

NOREPINEPHRINE EQUIVALENT DOSE CALCULATION

To allow standardized comparison among different vasopressors, we will calculate the norepinephrine equivalent (NEE) dose for each patient,. The NEE expresses the total vasopressor load in micrograms per kilogram per minute (µg/kg/min) of norepinephrine, enabling consistent assessment of shock severity regardless of the specific agent used. The calculation will be performed using the following updated formula:

NEE = norepinephrine + epinephrine + (0.1×dopamine) + (0.01×phenylephrine) + (2.5×vasopressin (U/min)) + (10×angiotensin II).

This approach allows integrating all vasoactive agents into a single continuous variable that reflects the overall hemodynamic support requirement.

Table 1S - Primary, secondary, and tertiary outcomes – overall treatment effect

Outcomes	Methylene blue	Usual care	Effect estimate [95%CI]	p-value
Primary composite outcome				
Initiation of vasopressor or an increase in vasopressor dose by at least 20% of baseline, or interruption of RRT session, or interruption of fluid removal	xx/xx (xx)	xx/xx (xx)	x.xx [x.xx - x.xx]	x.xxx
Individual components				
Initiation of vasopressor or an increase in vasopressor dose by at least 20% of baseline	xx/xx (xx)	xx/xx (xx)	x.xx [x.xx - x.xx]	-
Interruption of RRT session	xx/xx (xx)	xx/xx (xx)	x.xx [x.xx - x.xx]	-
Interruption of fluid removal	xx/xx (xx)	xx/xx (xx)	-	x.xxx
Secondary outcomes				
Hypotension during RRT	xx/xx (xx)	xx/xx (xx)	x.xx [x.xx - x.xx]	-
Ultrafiltration volume during RRT (mL)				
Maximum vasopressor dose within 24 hours (mcg/kg/minute)	x.xx (x.xx)	x.xx (x.xx)	MD[2] = x.xx [x.xx - x.xx]	-
Fluid balance within 24 hours (mL)				
Norepinephrine equivalent dose	xx/xx (xx)	xx/xx (xx)	x.xx [x.xx - x.xx]	
ICU mortality	xx/xx (xx)	xx/xx (xx)	x.xx [x.xx - x.xx]	-
Hospital mortality	xx/xx (xx)	xx/xx (xx)	x.xx [x.xx - x.xx]	-

Continue...

This is an open access article under the CC BY license (https://creativecommons.org/licenses/by/4.0/).

...continuation

Time to the primary outcome				
Cause of death				
Refractory septic shock	xx/xx (xx)	xx/xx (xx)	-	x.xxx
Multiple organ system dysfunction syndrome/acute respiratory failure	xx/xx (xx)	xx/xx (xx)	-	
New infectious episode	xx/xx (xx)	xx/xx (xx)	-	
Primary cardiac arrhythmia	xx/xx (xx)	xx/xx (xx)	-	
Hemorrhagic cerebrovascular disease	xx/xx (xx)	xx/xx (xx)	-	
Others	xx/xx (xx)	xx/xx (xx)	-	

95%CI - 95% confidence interval; RRT - renal replacement therapy; ICU - intensive care unit. (1) Combined outcome for 1. Initiation of vasopressor or an increase in vasopressor dose by at least 20% from baseline or 2. Interruption of renal replacement therapy session or 3. Switch to zero ultrafiltration at the request of the attending physician; (2) mean difference; (3) all models were adjusted for age, baseline vasopressor dose, and baseline mean arterial pressure. Results expressed as n/total (%) or n (%).

Table 2S - Baseline characteristics

Variável	Methylene blue (n = xx)	Usual care (n = xx)	Total (n = xxx)
Age (years)	xx (xx - xx)	xx (xx - xx)	xx (xx - xx
Sex (female)	xx/xx (xx%)	xx/xx (xx%)	xx/xx (xx%)
Weight (kg)	xx ± xx	xx ± xx	xx ± xx
SAPS 3	xx (xx - xx)	xx (xx - xx)	xx (xx - xx
SOFA D0	xx (xx - xx)	xx (xx - xx)	xx (xx - xx
Prior RRT during hospitalization			
Type of admission			
Medical	xx/xx (xx%)	xx/xx (xx%)	xx/xx (xx%)
Elective surgery	xx/xx (xx%)	xx/xx (xx%)	xx/xx (xx%)
Emergency surgery	xx/xx (xx%)	xx/xx (xx%)	xx/xx (xx%)
Admission source			
Ward	xx/xx (xx%)	xx/xx (xx%)	xx/xx (xx%)
Emergency department	xx/xx (xx%)	xx/xx (xx%)	xx/xx (xx%)
Operating room	xx/xx (xx%)	xx/xx (xx%)	xx/xx (xx%)
Comorbidities			
Diabetes mellitus	xx (xx)	xx (xx)	xx (xx)
Heart failure	xx/xx (xx%)	xx/xx (xx%)	xx/xx (xx%)
Chronic obstructive pulmonary disease	xx/xx (xx%)	xx/xx (xx%)	xx/xx (xx%)
Solid non-metastatic cancer	xx/xx (xx%)	xx/xx (xx%)	xx/xx (xx%)
Solid metastatic cancer	xx/xx (xx%)	xx/xx (xx%)	xx/xx (xx%)
Haematological malignancy	xx/xx (xx%)	xx/xx (xx%)	xx/xx (xx%)
Chronic kidney disease	xx/xx (xx%)	xx/xx (xx%)	xx/xx (xx%)
Cirrhosis			
HIV/AIDS			
Acute myocardial infarction			
Charlson Comorbidity Index	XXXXX	XXXXX	
Time from ICU admission to randomization			

SAPS 3 - Simplified Acute Physiology Score 3; SOFA - Sequential Organ Failure Assessment; RRT - renal replacement therapy; ICU - intensive care unit. Results expressed as median (interquartile), n (%) or standard deviation.

Table 3S - Characteristics of the hemodialysis session

Variable	Methylene blue	Usual care
	n = XX	n = XX
RRT characteristics		
Programmed RRT duration (min)	XXXXX	XXXXX
Programmed ultrafiltration rate	XXXXX	XXXXX
Programmed sodium profiling		
Programmed affluent temperature		
Vital signs immediately before initiation		
Systolic arterial pressure (mmHg)	XXXXX	XXXXX
Diastolic arterial pressure (mmHg)		
Mean arterial pressure (mmHg)	XXXXX	XXXXX
Heart rate (bpm)	XXXXX	XXXXX
Peripheral oxygen saturation (%)	XXXXX	XXXXX
PaO_2/FiO_2	XXXXX	XXXXX
Sedated at the start of hemodialysis		
Use of vasopressors (immediately before RRT)		
Noradrenaline	XXXXX	XXXXX
Dose (mcg/kg/min)	XXXXX	XXXXX
Epinephrine	XXXXX	XXXXX
Dose (mcg/kg/min)	XXXXX	XXXXX
Vasopressine	XXXXX	XXXXX
Dose (UI/min)	XXXXX	XXXXX
Dobutamine	XXXXX	XXXXX
Dose (mcg/kg/min)	XXXXX	XXXXX
Noradrenaline equivalent dose		
None	XXXXX	XXXXX

RRT - renal replacement therapy; PaO_2/FiO_2 - arterial partial pressure of oxygen/fraction of inspired oxygen. Results expressed as mean ± standard deviation or n (%),

Table 4S - Hemodynamic data during an off-renal replacement therapy session

Variable	Time point	Methylene blue	Usual care	Dif. Methylene blue-usual care [95%CI]
MAP	0	xx.x (xx.x), n = xx	xx.x (xx.x), n = xx	x.x [x.x to x.x]
	15	xx.x (xx.x), n = xx	xx.x (xx.x), n = xx	x.x [x.x to x.x]
	30	xx.x (xx.x), n = xx	xx.x (xx.x), n = xx	x.x [x.x to x.x]
	45	xx.x (xx.x), n = xx	xx.x (xx.x), n = xx	x.x [x.x to x.x]
	60	xx.x (xx.x), n = xx	xx.x (xx.x), n = xx	x.x [x.x to x.x]
	75	xx.x (xx.x), n = xx	xx.x (xx.x), n = xx	x.x [x.x to x.x]
	90	xx.x (xx.x), n = xx	xx.x (xx.x), n = xx	x.x [x.x to x.x]
	105	xx.x (xx.x), n = xx	xx.x (xx.x), n = xx	x.x [x.x to x.x]
	120	xx.x (xx.x), n = xx	xx.x (xx.x), n = xx	x.x [x.x to x.x]
	135	xx.x (xx.x), n = xx	xx.x (xx.x), n = xx	x.x [x.x to x.x]
	150	xx.x (xx.x), n = xx	xx.x (xx.x), n = xx	x.x [x.x to x.x]
	165	xx.x (xx.x), n = xx	xx.x (xx.x), n = xx	x.x [x.x to x.x]
	180	xx.x (xx.x), n = xx	xx.x (xx.x), n = xx	x.x [x.x to x.x]
	195	xx.x (xx.x), n = xx	xx.x (xx.x), n = xx	x.x [x.x to x.x]
	210	xx.x (xx.x), n = xx	xx.x (xx.x), n = xx	x.x [x.x to x.x]
	225	xx.x (xx.x), n = xx	xx.x (xx.x), n = xx	x.x [x.x to x.x]
	240	xx.x (xx.x), n = xx	xx.x (xx.x), n = xx	x.x [x.x to x.x]
SBP	0	xx.x (xx.x), n = xx	xx.x (xx.x), n = xx	x.x [x.x to x.x]
	15	xx.x (xx.x), n = xx	xx.x (xx.x), n = xx	x.x [x.x to x.x]
	30	xx.x (xx.x), n = xx	xx.x (xx.x), n = xx	x.x [x.x to x.x]
	45	xx.x (xx.x), n = xx	xx.x (xx.x), n = xx	x.x [x.x to x.x]
	60	xx.x (xx.x), n = xx	xx.x (xx.x), n = xx	x.x [x.x to x.x]
	75	xx.x (xx.x), n = xx	xx.x (xx.x), n = xx	x.x [x.x to x.x]
	90	xx.x (xx.x), n = xx	xx.x (xx.x), n = xx	x.x [x.x to x.x]
	105	xx.x (xx.x), n = xx	xx.x (xx.x), n = xx	x.x [x.x to x.x]
	120	xx.x (xx.x), n = xx	xx.x (xx.x), n = xx	x.x [x.x to x.x]
	135	xx.x (xx.x), n = xx	xx.x (xx.x), n = xx	x.x [x.x to x.x]
	150	xx.x (xx.x), n = xx	xx.x (xx.x), n = xx	x.x [x.x to x.x]
	165	xx.x (xx.x), n = xx	xx.x (xx.x), n = xx	x.x [x.x to x.x]
	180	xx.x (xx.x), n = xx	xx.x (xx.x), n = xx	x.x [x.x to x.x]
	195	xx.x (xx.x), n = xx	xx.x (xx.x), n = xx	x.x [x.x to x.x]
	210	xx.x (xx.x), n = xx	xx.x (xx.x), n = xx	x.x [x.x to x.x]
	225	xx.x (xx.x), n = xx	xx.x (xx.x), n = xx	x.x [x.x to x.x]
	240	xx.x (xx.x), n = xx	xx.x (xx.x), n = xx	x.x [x.x to x.x]

Continue...

...continuation

HR	0	xx.x (xx.x), n = xx	xx.x (xx.x), n = xx	x.x [x.x to x.x]
	15	xx.x (xx.x), n = xx	xx.x (xx.x), n = xx	x.x [x.x to x.x]
	30	xx.x (xx.x), n = xx	xx.x (xx.x), n = xx	x.x [x.x to x.x]
	45	xx.x (xx.x), n = xx	xx.x (xx.x), n = xx	x.x [x.x to x.x]
	60	xx.x (xx.x), n = xx	xx.x (xx.x), n = xx	x.x [x.x to x.x]
	75	xx.x (xx.x), n = xx	xx.x (xx.x), n = xx	x.x [x.x to x.x]
	90	xx.x (xx.x), n = xx	xx.x (xx.x), n = xx	x.x [x.x to x.x]
	105	xx.x (xx.x), n = xx	xx.x (xx.x), n = xx	x.x [x.x to x.x]
	120	xx.x (xx.x), n = xx	xx.x (xx.x), n = xx	x.x [x.x to x.x]
	135	xx.x (xx.x), n = xx	xx.x (xx.x), n = xx	x.x [x.x to x.x]
	150	xx.x (xx.x), n = xx	xx.x (xx.x), n = xx	x.x [x.x to x.x]
	165	xx.x (xx.x), n = xx	xx.x (xx.x), n = xx	x.x [x.x to x.x]
	180	xx.x (xx.x), n = xx	xx.x (xx.x), n = xx	x.x [x.x to x.x]
	195	xx.x (xx.x), n = xx	xx.x (xx.x), n = xx	x.x [x.x to x.x]
	210	xx.x (xx.x), n = xx	xx.x (xx.x), n = xx	x.x [x.x to x.x]
	225	xx.x (xx.x), n = xx	xx.x (xx.x), n = xx	x.x [x.x to x.x]
	240	xx.x (xx.x), n = xx	xx.x (xx.x), n = xx	x.x [x.x to x.x]
SpO_2	0	xx.x (xx.x), n = xx	xx.x (xx.x), n = xx	x.x [x.x to x.x]
	15	xx.x (xx.x), n = xx	xx.x (xx.x), n = xx	x.x [x.x to x.x]
	30	xx.x (xx.x), n = xx	xx.x (xx.x), n = xx	x.x [x.x to x.x]
	45	xx.x (xx.x), n = xx	xx.x (xx.x), n = xx	x.x [x.x to x.x]
	60	xx.x (xx.x), n = xx	xx.x (xx.x), n = xx	x.x [x.x to x.x]
	75	xx.x (xx.x), n = xx	xx.x (xx.x), n = xx	x.x [x.x to x.x]
	90	xx.x (xx.x), n = xx	xx.x (xx.x), n = xx	x.x [x.x to x.x]
	105	xx.x (xx.x), n = xx	xx.x (xx.x), n = xx	x.x [x.x to x.x]
	120	xx.x (xx.x), n = xx	xx.x (xx.x), n = xx	x.x [x.x to x.x]
	135	xx.x (xx.x), n = xx	xx.x (xx.x), n = xx	x.x [x.x to x.x]
	150	xx.x (xx.x), n = xx	xx.x (xx.x), n = xx	x.x [x.x to x.x]
	165	xx.x (xx.x), n = xx	xx.x (xx.x), n = xx	x.x [x.x to x.x]
	180	xx.x (xx.x), n = xx	xx.x (xx.x), n = xx	x.x [x.x to x.x]
	195	xx.x (xx.x), n = xx	xx.x (xx.x), n = xx	x.x [x.x to x.x]
	210	xx.x (xx.x), n = xx	xx.x (xx.x), n = xx	x.x [x.x to x.x]
	225	xx.x (xx.x), n = xx	xx.x (xx.x), n = xx	x.x [x.x to x.x]
	240	xx.x (xx.x), n = xx	xx.x (xx.x), n = xx	x.x [x.x to x.x]

Continue...

...continuation

Noradrenaline dose	0	xx.x (xx.x), n = xx	xx.x (xx.x), n = xx	x.x [x.x to x.x]
	15	xx.x (xx.x), n = xx	xx.x (xx.x), n = xx	x.x [x.x to x.x]
	30	xx.x (xx.x), n = xx	xx.x (xx.x), n = xx	x.x [x.x to x.x]
	45	xx.x (xx.x), n = xx	xx.x (xx.x), n = xx	x.x [x.x to x.x]
	60	xx.x (xx.x), n = xx	xx.x (xx.x), n = xx	x.x [x.x to x.x]
	75	xx.x (xx.x), n = xx	xx.x (xx.x), n = xx	x.x [x.x to x.x]
	90	xx.x (xx.x), n = xx	xx.x (xx.x), n = xx	x.x [x.x to x.x]
	105	xx.x (xx.x), n = xx	xx.x (xx.x), n = xx	x.x [x.x to x.x]
	120	xx.x (xx.x), n = xx	xx.x (xx.x), n = xx	x.x [x.x to x.x]
	135	xx.x (xx.x), n = xx	xx.x (xx.x), n = xx	x.x [x.x to x.x]
	150	xx.x (xx.x), n = xx	xx.x (xx.x), n = xx	x.x [x.x to x.x]
	165	xx.x (xx.x), n = xx	xx.x (xx.x), n = xx	x.x [x.x to x.x]
	180	xx.x (xx.x), n = xx	xx.x (xx.x), n = xx	x.x [x.x to x.x]
	195	xx.x (xx.x), n = xx	xx.x (xx.x), n = xx	x.x [x.x to x.x]
	210	xx.x (xx.x), n = xx	xx.x (xx.x), n = xx	x.x [x.x to x.x]
	225	xx.x (xx.x), n = xx	xx.x (xx.x), n = xx	x.x [x.x to x.x]
	240	xx.x (xx.x), n = xx	xx.x (xx.x), n = xx	x.x [x.x to x.x]
Adrenaline dose	0	xx.x (xx.x), n = xx	xx.x (xx.x), n = xx	x.x [x.x to x.x]
	15	xx.x (xx.x), n = xx	xx.x (xx.x), n = xx	x.x [x.x to x.x]
	30	xx.x (xx.x), n = xx	xx.x (xx.x), n = xx	x.x [x.x to x.x]
	45	xx.x (xx.x), n = xx	xx.x (xx.x), n = xx	x.x [x.x to x.x]
	60	xx.x (xx.x), n = xx	xx.x (xx.x), n = xx	x.x [x.x to x.x]
	75	xx.x (xx.x), n = xx	xx.x (xx.x), n = xx	x.x [x.x to x.x]
	90	xx.x (xx.x), n = xx	xx.x (xx.x), n = xx	x.x [x.x to x.x]
	105	xx.x (xx.x), n = xx	xx.x (xx.x), n = xx	x.x [x.x to x.x]
	120	xx.x (xx.x), n = xx	xx.x (xx.x), n = xx	x.x [x.x to x.x]
	135	xx.x (xx.x), n = xx	xx.x (xx.x), n = xx	x.x [x.x to x.x]
	150	xx.x (xx.x), n = xx	xx.x (xx.x), n = xx	x.x [x.x to x.x]
	165	xx.x (xx.x), n = xx	xx.x (xx.x), n = xx	x.x [x.x to x.x]
	180	xx.x (xx.x), n = xx	xx.x (xx.x), n = xx	x.x [x.x to x.x]
	195	xx.x (xx.x), n = xx	xx.x (xx.x), n = xx	x.x [x.x to x.x]
	210	xx.x (xx.x), n = xx	xx.x (xx.x), n = xx	x.x [x.x to x.x]
	225	xx.x (xx.x), n = xx	xx.x (xx.x), n = xx	x.x [x.x to x.x]
	240	xx.x (xx.x), n = xx	xx.x (xx.x), n = xx	x.x [x.x to x.x]

Continue...

...continuation

Vasopressin dose	0	xx.x (xx.x), n = xx	xx.x (xx.x), n = xx	x.x [x.x to x.x]
	15	xx.x (xx.x), n = xx	xx.x (xx.x), n = xx	x.x [x.x to x.x]
	30	xx.x (xx.x), n = xx	xx.x (xx.x), n = xx	x.x [x.x to x.x]
	45	xx.x (xx.x), n = xx	xx.x (xx.x), n = xx	x.x [x.x to x.x]
	60	xx.x (xx.x), n = xx	xx.x (xx.x), n = xx	x.x [x.x to x.x]
	75	xx.x (xx.x), n = xx	xx.x (xx.x), n = xx	x.x [x.x to x.x]
	90	xx.x (xx.x), n = xx	xx.x (xx.x), n = xx	x.x [x.x to x.x]
	105	xx.x (xx.x), n = xx	xx.x (xx.x), n = xx	x.x [x.x to x.x]
	120	xx.x (xx.x), n = xx	xx.x (xx.x), n = xx	x.x [x.x to x.x]
	135	xx.x (xx.x), n = xx	xx.x (xx.x), n = xx	x.x [x.x to x.x]
	150	xx.x (xx.x), n = xx	xx.x (xx.x), n = xx	x.x [x.x to x.x]
	165	xx.x (xx.x), n = xx	xx.x (xx.x), n = xx	x.x [x.x to x.x]
	180	xx.x (xx.x), n = xx	xx.x (xx.x), n = xx	x.x [x.x to x.x]
	195	xx.x (xx.x), n = xx	xx.x (xx.x), n = xx	x.x [x.x to x.x]
	210	xx.x (xx.x), n = xx	xx.x (xx.x), n = xx	x.x [x.x to x.x]
	225	xx.x (xx.x), n = xx	xx.x (xx.x), n = xx	x.x [x.x to x.x]
	240	xx.x (xx.x), n = xx	xx.x (xx.x), n = xx	x.x [x.x to x.x]
Dobutamine dose	0	xx.x (xx.x), n = xx	xx.x (xx.x), n = xx	x.x [x.x to x.x]
	15	xx.x (xx.x), n = xx	xx.x (xx.x), n = xx	x.x [x.x to x.x]
	30	xx.x (xx.x), n = xx	xx.x (xx.x), n = xx	x.x [x.x to x.x]
	45	xx.x (xx.x), n = xx	xx.x (xx.x), n = xx	x.x [x.x to x.x]
	60	xx.x (xx.x), n = xx	xx.x (xx.x), n = xx	x.x [x.x to x.x]
	75	xx.x (xx.x), n = xx	xx.x (xx.x), n = xx	x.x [x.x to x.x]
	90	xx.x (xx.x), n = xx	xx.x (xx.x), n = xx	x.x [x.x to x.x]
	105	xx.x (xx.x), n = xx	xx.x (xx.x), n = xx	x.x [x.x to x.x]
	120	xx.x (xx.x), n = xx	xx.x (xx.x), n = xx	x.x [x.x to x.x]
	135	xx.x (xx.x), n = xx	xx.x (xx.x), n = xx	x.x [x.x to x.x]
	150	xx.x (xx.x), n = xx	xx.x (xx.x), n = xx	x.x [x.x to x.x]
	165	xx.x (xx.x), n = xx	xx.x (xx.x), n = xx	x.x [x.x to x.x]
	180	xx.x (xx.x), n = xx	xx.x (xx.x), n = xx	x.x [x.x to x.x]
	195	xx.x (xx.x), n = xx	xx.x (xx.x), n = xx	x.x [x.x to x.x]
	210	xx.x (xx.x), n = xx	xx.x (xx.x), n = xx	x.x [x.x to x.x]
	225	xx.x (xx.x), n = xx	xx.x (xx.x), n = xx	x.x [x.x to x.x]
	240	xx.x (xx.x), n = xx	xx.x (xx.x), n = xx	x.x [x.x to x.x]

95%CI - 95% confidence interval; MAP - mean arterial pressure; SBP - systolic blood pressure; HR - heart rate; SpO_2 - blood oxygen saturation.

Table 5S - Adverse events according to the group

Variable	Methylene blue	Usual care	Total	p value
	n = XX	n = XX	n = XX	
Adverse events	xx/xx (xx,x)	xx/xx (xx,x)	xx/xx (xx,x)	x.xxx
Acute myocardial infarction				
Occurrence of hemolytic anemia				
Cardiopulmonary arrest during the session				
Nausea and vomiting				
Malignant hyperthermia				
Precordial pain				
Hypertension				
Worsening of gas exchange				
Serious adverse events	xx/xx (xx,x)	xx/xx (xx,x)	xx/xx (xx,x)	x.xxx
XXXXXX	xx/xx (xx,x)	xx/xx (xx,x)	xx/xx (xx,x)	x.xxx
XXXXXX	xx/xx (xx,x)	xx/xx (xx,x)	xx/xx (xx,x)	x.xxx
XXXXXX	xx/xx (xx,x)	xx/xx (xx,x)	xx/xx (xx,x)	x.xxx

Results expressed as n (%).

Table 6S - Adherence to study protocol

Variable	Methylene blue	Usual care
	n = XX	n = XX
Received no intervention	xx/xx (xx,x)	xx/xx (xx,x)
Received only bolus		
Received bolus and partial infusion during RTT		
Received bolus and infusion during the whole RTT		
No bolus, only infusion during RTT		

RRT - renal replacement therapy. Results expressed as n/total (%).

Table 7S - Data collection schedule

Variable	Baseline ICU admission	Before the RTT session	T0-T6 RTT	24-hour assessments	ICU discharge	Hospital discharge
Eligibility criteria	X					
Informed consent	X					
Weight	X					
Sex at birth	X					
Ethnicity	X					
Age	X					
Comorbidities	X					
SAPS 3	X					
Randomization		X				
SOFA score		X				
Type of admission		X				
Sedation		X				
Mechanical ventilation		X				
Programmed ultrafiltration rate		X				
Programmed sodium profiling		X				
Programmed affluent temperature		X				
Hemodynamic data		X				
MAP		X	X			
SBP		X	X			
DBP		X	X			
HR		X	X			
FiO_2		X	X			
SpO_2		X	X			
PaO_2/FiO_2		X	X	X		
Vasopressor dose		X	X	X		
RTT session fluid balance			X			
Adverse events			X	X	X	X
24-hour fluid balance				X		
Maximum vasopressor dose				X		
Death					X	X

ICU - intensive care unit; RRT - renal replacement therapy; SOFA - Sequential Organ Failure Assessment; MAP – mean arterial pressure; SBP - systolic blood pressure; DBP - diastolic blood pressure; HR - heart rate; FiO_2 - arterial partial pressure of oxygen; SpO_2 - blood oxygen saturation; PaO_2 - arterial partial pressure of oxygen.
